# Dexpramipexole Attenuates White Matter Injury to Facilitate Locomotion and Motor Coordination Recovery via Reducing Ferroptosis after Intracerebral Hemorrhage

**DOI:** 10.1155/2022/6160701

**Published:** 2022-08-04

**Authors:** Bo Wang, Xuyang Zhang, Jun Zhong, Shi Wang, Chao Zhang, Mingxi Li, Quan Hu, Shuhong Wang, Lin Chen, Weixiang Chen, Hongfei Ge, Hua Feng

**Affiliations:** ^1^Department of Neurosurgery and key Laboratory of Neurotrauma, Southwest Hospital, Third Military Medical University (Army Medical University), 400038 Chongqing, China; ^2^Department of Neurosurgery, No. 96603 Unit Hospital of People's Liberation Army of China, 418000 Huaihua, Hunan, China; ^3^Department of Emergency, Affiliated Hospital of Zunyi Medical University, 563003 Zunyi, Guizhou, China; ^4^Department of Neurosurgery, General Hospital of Xinjiang Military Region, 830000 Urumqi, Xinjiang, China

## Abstract

Deciphering the factors causing damage to white matter fiber bundles and exploring new strategies to alleviate white matter injury (WMI) is a promising treatment to improve neurological impairments after intracerebral hemorrhage (ICH). Ferroptosis usually occurs at perihematomal region and contributes to neuronal death due to reactive oxygen species (ROS) production. Dexpramipexole (DPX) easily crosses the blood brain barrier (BBB) and exerts antioxidative properties by reducing ROS production, while the role of DPX in ferroptosis after ICH remains elusive. Here, our results indicated that ferroptosis played a significant role in WMI resulting from iron and ROS accumulation around hematoma. Further evidence demonstrated that the administration of DPX decreased iron and ROS deposition to inhibit ferroptosis at perihematomal site. With the inhibition of ferroptosis, WMI was alleviated at perihematomal site, thereafter promoting locomotion and motor coordination recovery in mice after ICH. Subsequently, the results showcased that the expression of glutathione peroxidase 4 (GPX4) and ferroptosis suppressing protein 1 (FSP1) was upregulated with the administration of DPX. Collectively, the present study uncovers the underlying mechanism and elucidates the therapeutic effect of DPX on ICH, and even in other central nervous system (CNS) diseases with the presence of ferroptosis.

## 1. Introduction

Intracerebral hemorrhage (ICH), accounting for 15-20% of strokes, is a life-threating disease, and nearly each survivor suffers from life-long neurological deficits [1–3]. The predilection site of ICH is basal ganglia, where enriches white matter fiber bundles [4], whose integrity is a determinant for functional recovery in patients with ICH [5]. Thus, reducing white matter injury (WMI) to maintain the integrity of white matter fiber bundles is a feasible strategy to improve neurological impairments in patients with ICH. Meanwhile, our previous studies demonstrate that several interventions are capable of palliating WMI to promote functional recovery such as lithium [6], cattle encephalon glycoside and ignotin [7], and ambroxol [3]. Subsequently, our retrospective clinical study shows that ICH patients underwent the image-guided para-corticospinal tract approach which exhibit better functional recovery based on the protection of compressed or residual white matter fiber bundles [5]. However, the therapeutic effect is still far from ideal. Hence, deciphering the factors causing damage to white matter fiber bundles and exploring therapeutic strategy might promote functional recovery after ICH.

Ferroptosis, a form of regulated cell death, is characterized by the iron-induced accumulation of lipid reactive oxygen species (ROS) that leads to intracellular oxidative stress [8]. Previous studies have indicated that ferroptosis usually occurs at perihematomal site and contributes to neuronal death due to ROS production and lipid peroxidation after ICH [9–11]. Oligodendrocytes, which are mainly responsible for myelination in the central nervous system (CNS) [12, 13], are especially rich in unsaturated fatty acid [4] and susceptible to ferroptosis-induced damage [14]. Our previous study indicates that ferroptosis plays a pivotal role in damaging oligodendrocytes to exaggerate WMI and the application of ferrostatin-1 palliates ferroptosis to facilitate functional recovery after spinal cord injury (SCI) [15]. Thereafter, exploring more treatments targeting WMI is of great significance.

Dexpramipexole (DPX) might serve as a candidate alleviating WMI to promote functional recovery after ICH. DPX is an R-isomer of antiparkinson drug pramipexole, but with very low affinity for dopamine receptors [16], which makes it unlikely to be associated with dopaminergic side effects, such as orthostatic hypotension and hallucination [17]. Hence, DPX is safe and well tolerated in the treatment of Parkinson's disease (PD). Previous studies have demonstrated that DPX holds the unique capability of binding to mitochondrial F1Fo-ATP synthase activator, thereafter improving mitochondrial efficiency by increasing ATP synthesis and reducing oxygen (O_2_) consumption to support neuronal bioenergetics [18–20]. Furthermore, organic cation/carnitine transporter (OCTN) 1 and 2 could transport DPX across the blood brain barrier (BBB) [21], and DPX potentially accumulates in the brain parenchyma (brain/plasma ratio > 15) when administrating DPX [19, 22], indicating that DPX could serve as a candidate to facilitate neuronal survival. Additionally, studies have demonstrated that DPX exerts antioxidative properties by reducing the production of ROS without causing side effects [22, 23], implying that DPX might protect oligodendrocytes from ferroptosis by decreasing ROS around perihematomal region after ICH.

Here, we posited that DPX facilitated oligodendrocytes survival to maintain the integrity of white matter fiber bundles through reducing ferroptosis, thereafter expediting functional recovery. The aim of the present study is to clarify the neuroprotective effect of DPX on WMI and to uncover the underlying mechanism after ICH.

## 2. Materials and Methods

### 2.1. Animals and Experimental Design

All experimental procedures were supervised by the Ethics Committee of the Southwest Hospital, Third Military Medical University, and conformed to the guidelines of the National Institutes of Health Guide for the Care and Use of Laboratory Animals (approval no. AMUWEC2020761). A total of 156 adult C57BL/6 mice (male, 24-26 g, 9-10 weeks, 145 used for experiments and 11 died during experiments) were acquired from laboratory animal center of the Third Military Medical University for ICH model.

### 2.2. Mouse ICH Model and Groups

Before anesthesia, the weight of each mouse was collected. Then, mice were anesthetized with 2% isoflurane/air mixture (2-3 l/min) and fixed onto a stereotactic instrument (RWD, Shenzhen, China). The mouse ICH model was established as previously described [24]. In brief, a total of 26 *μ*l autologous blood from tail vein were collected and injected into basal ganglia (from bregma: 2.0 mm lateral, 0.8 mm anterior, and 3.0 mm ventral) with a speed of 2 *μ*l/min using a sterile Hamilton syringe (Imboden, Canton, Switzerland) after an incision was made on the scalp and a cranial hole was drilled. The needle was fixed for at least 5 minutes to prevent the back-flow when injection was finished. Body temperatures were maintained at 37 ± 0.3°C using a feedback-controlled heating system (Zhongshi, Inc., Beijing, China) during surgery. Then, mice were given free access to food and water under the condition of constant photoperiod (12-h light/dark cycle), temperature (22-25°C) and moisture (55-60%) before and after surgery. For the dosage of DPX administration in the present study was referred to the previous study that was 10 mg/kg [25]. At the same time, the lower and higher dosages (5 and 20 mg/kg) were administrated to optimize the feasible dosage for the treatment of ICH.

These mice were randomly divided into 3 groups and the following experiments were performed: Sham (0.9% NaCl), ICH (ICH+0.9% NaCl), and ICH + DPX (ICH + DPX). For Sham group, mice were only received needle insertion. For ICH group, mice were received 26 *μ*l autologous blood. For ICH + DPX group, mice were further randomly divided into three groups with different dosages (5, 10, or 20 mg/kg). DPX (MedChemExpress, Shanghai, China) was dissolved in 0.9% NaCl and were intraperitoneally injected. Mice in other groups intraperitoneally received the same volume of 0.9% NaCl as that in the group of ICH+20 mg/kg DPX.

### 2.3. Behavioral Tests

#### 2.3.1. Open Field Test

The open field test (OFT) was performed to assess locomotion recovery in mice [26]. The field was equally divided into four chambers, with each one (50 cm × 50 cm and 50 cm high wall) adjoining each other but isolated. Each mouse was placed in the same chamber for 5 min, and video was recorded. Then, the chamber was cleaned up for the next test. The video was then analyzed by two independent examiners blinded to the experimental groups to measure the whole distance of free movement and mean velocity using a software (ViewPoint Behaviour Technology, Lyon, France).

#### 2.3.2. Beam Walking Test

The beam walking test (BWT) was conducted to measure the motor coordination after ICH [27]. Briefly, a 100-cm long, 1-cm thick wooden beam was set up at 50 cm above the ground. Before surgery, mice were allowed to walking from one end of the beam to another one for several times until they could fluently pass. After surgery, each mouse was allowed to walk from one side to the other side 3 times during each time point, and video was recorded. Then, the times of hindlimb flip from the beam (hindlimb fault) due to disability during 3 times traveling were calculated from the video by two independent examiners blinded to the experimental groups.

#### 2.3.3. Forelimb Muscle Strength Test

Forelimb muscle strength was measured by a grip strength meter (Laboratory Enterprises, Nasik, India), which consisted of a steel wire grid (8 × 8 cm) connected to an isometric force transducer. Each mouse was tested three times, and average value was recorded. The test was performed by two investigators blinded to the group assignment.

#### 2.3.4. Basso Mouse Score (BMS)

Basso mouse score (BMS) is used for evaluating locomotion and coordination. Each mouse was place in an open field for scoring each time. The scoring system is ranging from 0 to 9: 0 points, no ankle movement; 1 point, slight ankle movement; 2 points, extensive ankle movement; 3 points, plantar placement with or without weight support; 4 points, occasional plantar stepping; 5 points, frequent or consistent plantar stepping, no coordination; 6 points, frequent or consistent plantar stepping, some coordination, paws parallel at initial contact; 7 points, frequent or consistent plantar stepping, mostly coordinated, paws parallel at initial contact and rotated and lift of; 8 points, frequent or consistent plantar stepping, mostly coordinated, paws parallel at initial contact and lift of, and mild trunk instability; and 9 points, frequent or consistent plantar stepping, mostly coordinated, paws parallel at initial contact and lift of, and normal trunk stability and tail always up. The test was performed by two investigators blinded to the group dividing. Average score from two investigator was recorded.

### 2.4. Transmission Electron Microscopy (TEM)

TEM was performed to visualized ultrastructural of white matter fiber bundles in different groups, as previously described [15]. In brief, the samples (about 1 mm^3^ perihematoma) were incubated in 1.25% glutaraldehyde overnight after perfusion. Then, specimens were post-fixed with 1.25% glutaraldehyde at least 3 days in 4 °C. Afterward, the tissues were rinsed 3 times and fixed with 1% citric acid (OsO4) for 2 h. And, uranyl acetate was used for redyeing, while gradient acetone was used for dehydration. Thereafter, an ultramicrotome (EM UC7, Leica, IL, USA) was used for slicing before the samples were infiltrated with propylene oxide and embedded by epoxy. Subsequently, the samples were observed by a transmission electron microscope (Hitachi HT7700, Tokyo, Japan). At least three independent samples per group were used for analysis. The G-ratios of the myelinated fibers were calculated as the ratio of the diameter of axon to the diameter of axon and myelin sheath. The figures acquired from TEM were randomly selected for G-ratio calculation. At least 70 myelinated fibers from each group were calculated using an Image J software (ImageJ 1.8, NIH, USA) by two independent examiners blinded to the experimental groups.

### 2.5. Hematoxylin and Eosin (HE) Staining

HE staining was used to evaluate the hematoma size in order to reflect the injury and necrosis degree [28]. Briefly, brain sections were sliced by a freezing microtome, washed with distilled water, stained with Hematoxylin staining solution for 10 min, then rinsed 3 times with distilled water, differentiated in 0.1% hydrochloric acid-ethanol for 25 s, blued in phosphate-buffered solution (PBS) for 45 min followed by 95% alcohol washing for 5 s, then counterstained with Eosin staining solution for 1 min, dehydrated with 95% alcohol, cleared with xylene, and finally mounted. The samples were observed and analyzed using an image processing system (Case viewer, 3DHISTEC, Budapest, Hungary). Infarct volume was calculated by hemorrhagic area of all sections and multiplying by slice thickness. All experiments and analysis were conducted by individual investigator blinded to treatment groups.

### 2.6. Dihydroethidium (DHE) Staining

DHE staining was performed to indicate the ROS accumulation. Briefly, brain sections were incubated in 10 *μ*M dihydroethidium (DHE) solution (MedChemExpress, Shanghai, China) for 1 h in dark at 37 °C. Afterward, the sections were washed with PBS for 3 times. Thereafter, 4-6-diamidino-2-phenylindole (DAPI) was used to counterstained cell nuclei for 5 min at room temperature. Subsequently, the samples were mounted with neutral balata and observed with the fluorescent microscope (Carl Zeiss, Weimar, Germany). The positive area was calculated using an Image J software (ImageJ 1.8, NIH, USA).

### 2.7. Perl's Blue Staining

Perl's blue staining was used to reflect iron deposition. In brief, brain sections (25 *μ*m) were washed with distilled water. Then, the Perl's blue kit (Solarbio, Beijing, China) was administrated to stain samples. First, the same volume of reagent A1 and reagent A2 was mixed; then, the sections were incubated in the solution for 20 min. After the specimens were washed using distilled water, the reagent B was used to stain the sections for 5 min. Then, the slices were mounted with neutral balata after washed with distilled water for 2-5 seconds. Images were captured using a light microscope (Axio lab, ZEISS, Weimar, Germany) and analyzed by an Image J software (ImageJ 1.8, NIH, USA).

### 2.8. Malondialdehyde (MDA) Tests

MDA tests were conducted to measure the level of lipid peroxidation by an MDA kit R&D Systems, USA). Briefly, the fitted standard curve was established to determine the levels of MDA concentration. Then, the ipsilateral brain tissues around hematoma on day 7 were collected for lysis and centrifuge. Supernatants were firstly underwent acidification and then centrifuged for 3 times. Afterward, the samples were mixed with TBARS reagent for 2-3 h at 40-50 °C. The optic density (OD) value was determined at 532 nm using a spectrophotometer (Varioskan Flash, Thermo Scientific, Waltham, MA, USA); then, the MDA concentration was calculated by the fitted standard curve.

### 2.9. Immunostaining

Brain sections were immersed in 0.3% Triton-X 100 (Sigma-Aldrich, St. Louis, MO) in PBS for 30 min at room temperature. Then, sections were incubated in the following primary antibodies overnight at 4 °C after blocked with 5% bovine serum album (BSA, Sigma-Aldrich, St. Louis, MO, German): rabbit anti-myelin basic protein (MBP; 1 : 250, Boster, Wuhan, China), mouse anti-Alzheimer precursor protein A4 (APP; 1 : 250, Sigma-Aldrich, St. Louis, MO, German), mouse anti-MBP (1 : 250, Abcam, Abcam, Cambridge, UK), rabbit anti-ferroptosis suppressing protein 1 (FSP1; 1 : 250, Proteintech, Wuhan, China), and rabbit anti-glutathione peroxidase 4 (GPX4; 1 : 1000, Boster, Wuhan, China). After washed with PBS for 3 times, the samples were incubated in Alexa Fluor 488- or 555-conjugated secondary antibodies (1 : 1000, Thermo Fisher Scientific, Waltham, MA, USA). Subsequently, the nuclei were counterstained with DAPI. Afterward, the sections were sealed with anti-fluorescent decay reagent (Boster, Wuhan, China), visualized by a confocal microscope (LSM880, Carl Zeiss, Weimar, Germany), and analyzed by an Image J software (ImageJ 1.8, NIH, USA).

### 2.10. Western Blot

Brain tissues around the hematoma were collected after decapitation post-ICH on day 7. The tissue lysates were obtained after centrifuge, and the protein concentration of each sample was determined using a bicinchoninic acid (BCA) method (Beyotime, Shanghai, China). A total of 50 *μ*g protein were separated by 10% or 12.5% SDS-PAGE under reducing conditions and electro-blotted to polyvinylidene difluoride (PVDF, Roche, IN, USA) membranes. Thereafter, the membranes were incubated in 5% (w/v) non-fat dry milk (Beyotime, Shanghai, China) in Tris-HCl buffer solution + Tween (TBST) at room temperature for 2 h. Afterward, the membranes were incubated in primary antibodies at 4 °C overnight: rabbit anti-myelin basic protein (MBP; 1 : 1000, Boster, Wuhan, China), rabbit anti-neurofilament heavy (NF200; 1 : 1000, Boster, Wuhan, China), rabbit anti-myelin basic protein degraded (dMBP; 1 : 1000, Mybiosource, San Diego, CA, USA), rabbit anti-ferroptosis suppressing protein 1 (FSP1; 1 : 1000, Proteintech, Wuhan, China), rabbit anti-glutathione peroxidase 4 (GPX4; 1 : 1000, Boster, Wuhan, China), and rabbit anti-*β*-Tubulin (1 : 2000, Proteintech, Wuhan, China). Then, after washing with TBST for 3 times, the membranes were immersed in related secondary antibodies (1 : 5000, Boster, Wuhan, China) at room temperature for 2 h. The optic density was visualized using an imaging system (Evolution-Capt Edge, Vilber, France) with a Western blot chemiluminescence kit (Thermo Fisher Scientific, Waltham, MA, USA). Densitometric measurement of each membrane was performed using Image Lab™ software (Bio-Rad, California, USA).

### 2.11. Statistical Analysis

All data were presented as mean ± SEM. The statistical analyses were performed using SPSS 18.0 software (SPSS, Inc., Chicago, IL, United States). The Kaplan-Meier was used to observe the survival curves of among different groups, and analyzed using the Log rank test. Behavioral data collected at repeating time points were analyzed using two-way analysis of variance (ANOVA), followed by Turkey's post hoc test. For data with a single time point, multiple comparisons were performed by one-way ANOVA, and then multiple comparisons were performed using Turkey's post hoc test in case of the data with a normal distribution using a Shapiro-Wilk normality test. A *p* < 0.05 was considered to be statistically significant.

## 3. Results

### 3.1. The Administration of DPX Facilitated Locomotion and Motor Coordination Recovery by Decreasing Hematoma Volume in Mice after ICH

**Figure 1:**
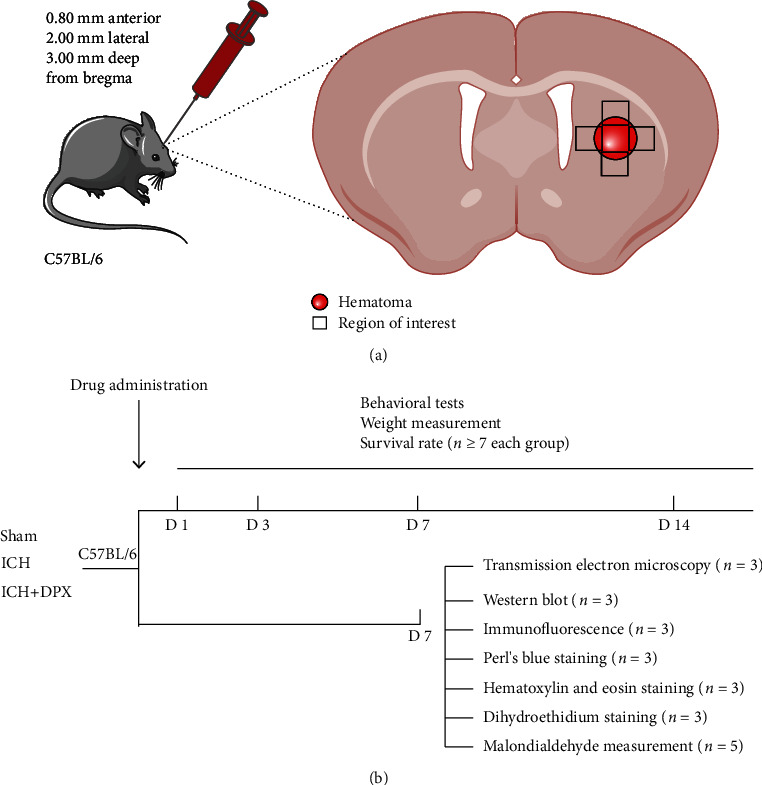
Schematic illustration of mouse ICH model and experimental design. (a) Details of ICH model and the black squares illustrating the region of interesting for further analyses. (b) Flow chart of group assignment and experimental arrangements.

To evaluate the role of DPX on functional recovery and the underlying mechanism after ICH in mice, the mouse ICH model was firstly established as shown in Figure 1(a). Then, mice were designated into five groups: Sham, ICH, ICH + DPX 5 mg/kg, ICH+ DPX 10 mg/kg, and ICH + DPX 20 mg/kg. The survival rate, behavioral tests, and weight measurement were performed to determine the timepoint and dosage for future research on days 1, 3, 7, and 14 (Figure 1(b)). Thereafter, various examinations were implemented on selected day, that is, on day 7 in the present work (Figure 1(b)).

**Figure 2:**
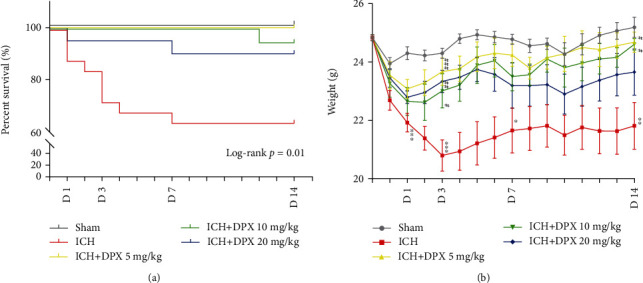
The administration of DPX facilitated survival and weight gain after ICH in mice. (a) Survival rate of mice in each group. *n* = 21 in Sham group, *n* = 25 in ICH group, *n* = 20 in ICH + DPX 5 mg/kg group, *n* = 19 in ICH + DPX 10 mg/kg group, and *n* = 20 in ICH + DPX 20 mg/kg group; Log rank test Chi square = 22.92; *p* = 0.01; Log rank test. (b) Weight change of mice in each group. n =9 in Sham group, *n* = 14 in ICH group, *n* = 10 in ICH + DPX 5 mg/kg group, *n* = 9 in ICH + DPX 10 mg/kg group, and *n* = 11 in ICH + DPX 20 mg/kg group; F (4, 48) = 6.897 on day 1; F (4, 48) = 8.749 on day 3; F (4, 48) = 3.472 on day 7; and F (4, 48) = 4.452 on day 14; ∗*p* < 0.05, ∗∗*p* < 0.01, ∗∗∗*p* < 0.001 vs. Sham group; ^#^*p* < 0.05, ^##^*p* < 0.01, ^###^*p* < 0.001 vs. ICH group; two-way ANOVA, followed by Turkey's post hoc test.

First, the survival rate of mice in each group was assessed using survival curve. The curve indicated that the administration of DPX significantly increased the survival rate, compared with ICH group (Figure 2(a)). And the mice received 5 mg/kg and 10 mg/kg DPX showed higher surviving percentage than that in ICH group (Figure 2(a)). Then, the weight of mice with the administration of DPX was heavier than that in the ICH group from days 3 to 14, especially the weight of mice lowered to the trough on day 3 in ICH group (Figure 2(b)). Interestingly, the weight of mice in ICH + DPX 20 mg/kg group showed a downward trend and that in ICH + DPX 10 mg/kg group presented a raising tendency (Figure 2(b)). Meanwhile, the weight of mice in ICH + DPX 5 mg/kg group exhibited the best condition among all the groups (Figure 2(b)).

**Figure 3:**
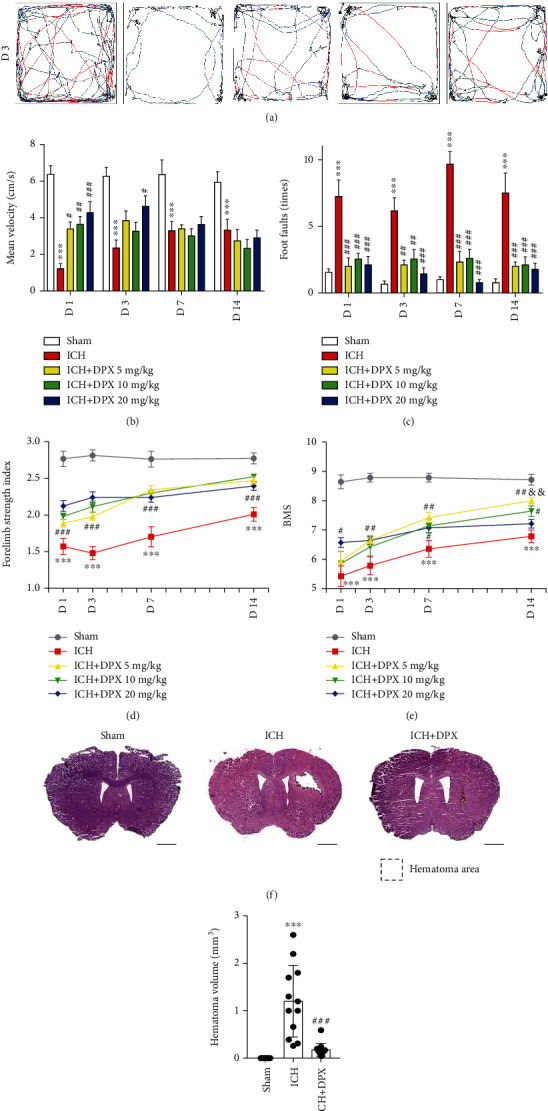
The application of DPX facilitated locomotion and coordination recovery through minimizing hematoma volume after ICH. (a) Representative trajectory of mice during open field test. Blue square: the starting point. Yellow circle: the ending point. Black trajectory: velocity < 7 cm/s. Blue trajectory: velocity > 7 but <25 cm/s. Red trajectory: velocity > 25 cm/s. (b) Mean velocity of mice in each group in open field test. *n* = 9 in Sham group, *n* = 8 in ICH group, *n* = 8 in ICH + DPX 5 mg/kg group, *n* = 8 in ICH + DPX 10 mg/kg group, and *n* = 7 in ICH + DPX 20 mg/kg group; F (4, 35) = 19.15; ^∗∗∗^*p* < 0.001 vs. Sham group; ^#^*p* < 0.05, ^##^*p* < 0.01, and ^###^*p* < 0.001 vs. ICH group; two-way ANOVA, followed by Turkey's post hoc test. (c) Beam walking test of mice in each group. *n* = 9 in Sham group, *n* = 12 in ICH group, *n* = 9 in ICH + DPX 5 mg/kg group, *n* = 9 in ICH + DPX 10 mg/kg group, *n* = 9 in ICH + DPX 20 mg/kg group; F (4, 43) = 34.47; ^∗∗∗^*p* < 0.001 vs. Sham group; ^##^*p* < 0.01 and ^###^*p* < 0.001 vs. ICH group; two-way ANOVA, followed by Turkey's post hoc test. (d) Forelimb muscle strength of mice in each group. Data was shown by mN/per gram weight. *n* = 7 in each group; F (4, 30) = 61.97; ^∗∗∗^*p* < 0.001 vs. Sham group; ^###^*p* < 0.001 vs. ICH group; two-way ANOVA, followed by Turkey's post hoc test. (e) Basso mouse score of mice in each group. *n* = 7 in each group; F (4, 30) = 30.19; ^∗∗∗^*p* < 0.001 vs. Sham group; ^#^*p* < 0.05 and ^##^*p* < 0.01 vs. ICH group; ^&&^*p* < 0.01 vs. ICH+20 mg/kg DPX group; two-way ANOVA, followed by Turkey's post hoc test. (f) Typical HE staining images presenting the hematoma size in each group. Scale bars: 1000 *μ*m. (g) Quantification of hematoma size from (f). *n* = 7 in Sham group, *n* = 12 in ICH group, *n* = 13 in ICH + DPX group; F (2, 29) = 20.08; ^∗∗∗^*p* < 0.001 vs. Sham group; ^###^*p* < 0.001 vs. ICH group. One-way ANOVA, followed by Turkey's post hoc test.

Then, the OFT was performed to evaluate locomotion recovery in mice post-ICH. The results showed that the mean velocity of mice in ICH group was significantly decreased at the acute phase from days 1 to 3, with a recovery in the following days after ICH (Figures 3(a) and 3(b)), while the mean velocity of mice administrated DPX (5, 10, and 20 mg/kg) obviously increased compared with that of mice in ICH group on day 1 (Figures 3(a) and 3(b)). Meanwhile, the mean velocity of mice in ICH + DPX 20 mg/kg group was evidently increased than that in ICH group, but no significant difference was observed among ICH + DPX 5 mg/kg group, ICH + DPX 10 mg/kg group, and ICH group on day 3 (Figures 3(a) and 3(b)). And the results demonstrated that the mean velocity of mice administrated DPX (5, 10, and 20 mg/kg) showed no obvious difference in comparison with that of ICH mice (Figures 3(a) and 3(b)). Afterward, the BWT was applied to certify the effect of DPX on motor coordination in mice after ICH. The results indicated that the number of foot faults of mice with the administration of DPX (5, 10, and 20 mg/kg) was markedly decreased, while that was clearly increased in mice without the application of DPX (ICH group) on days 1, 3, 7, and 14 after ICH (Figure 3(c)). Subsequently, the forelimb muscle strength test exhibited the same tendency as BMT (Figure 3(d)). Intriguingly, the BMS showed that mice in ICH + DPX 20 mg/kg group exhibited higher score on day 1, while no obvious benefit was observed than other two dosages (Figure 3(e)), whereas the BMS of mice in ICH + DPX 5 mg/kg group presented an increased trend from day 1 to 14 and showed a significant difference between ICH + DPX 5 mg/kg group and ICH + DPX 20 mg/kg group on day 14 (Figure 3(e)). At the same time, the administration of 10 mg/kg DPX showed moderate benefit, compared to the other two dosages (Figure 3(e)).

The above findings illustrated that the application of DPX reduced survival rate, increased weight, and promoted locomotion and motor coordination recovery, implying that DPX might minimize hematoma injury. Consider the administration of 5 mg/kg DPX exhibited a slighter influence on the weight and almost the same effect on BMT in mice received 10 mg/kg or 20 mg/kg DPX after ICH. Furthermore, the administration of 5 mg/kg DPX represented an elevated tendency from days 1 to 14 and exhibited higher score in BMS than other two dosages from day 7. Thence, the dosage of 5 mg/kg was used in the further research. Meantime, the timepoint was on day 7 post-ICH. Subsequently, the hematoma size was determined using HE staining on day 7, and the images delineated that the volume of hematoma was significantly decreased with the administration of 5 mg/kg DPX (Figures 3(f) and 3(g)). Together, these results illustrated that the administration of DPX promoted locomotion and motor coordination recovery through reducing hematoma volume after ICH in mice.

### 3.2. The Application of DPX Reduced WMI around Hematoma in Mice after ICH

**Figure 4:**
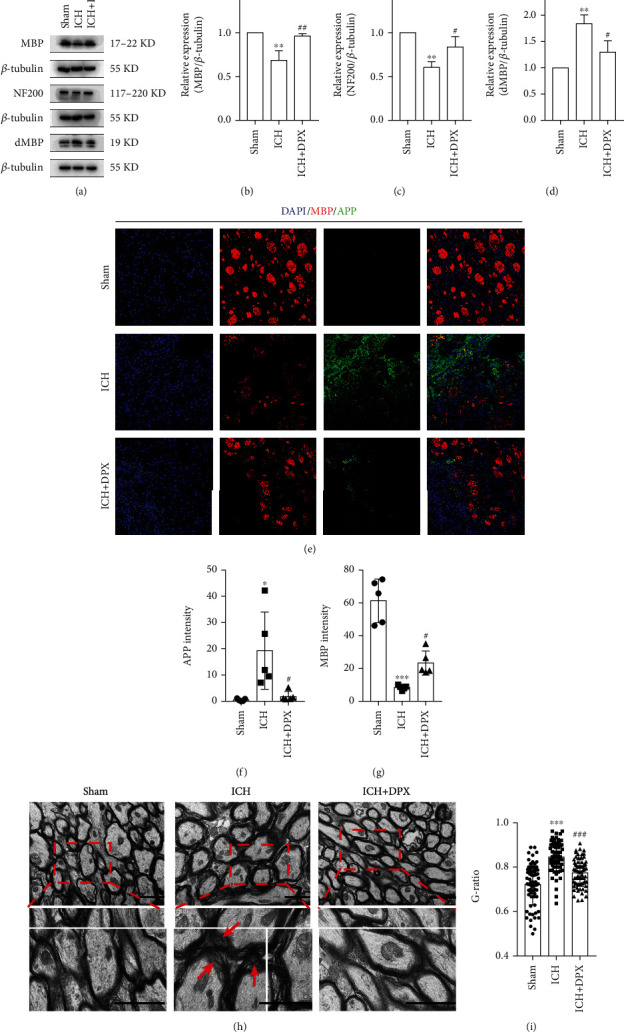
The application of DPX reduced white matter damage. (a) Immunoblot bands depicting the expression of MBP, NF200, and dMBP in each group. *β*-Tubulin was served as an internal control. (b)–(d) Semi-quantification of the expression of MBP (e), NF200 (f) and dMBP (g). *n* = 3 in each group; F (2, 6) = 20.87 for MBP; F (2, 6) = 18.94 for NF200; F (2, 6) = 21.18 for dMBP; ^∗^*p* < 0.05 and ^∗∗^*p* < 0.01 vs. Sham group; ^#^*p* < 0.05 and ^###^*p* < 0.001 vs. ICH group; one-way ANOVA, followed by Turkey's post hoc test. (e) Double immunostaining of MBP (red) and APP (green). Scale bars: 50 *μ*m. (f), (g) Semiquantification of the optic intensity of APP (f) and MBP (g). *n* = 5 in each group; F (2, 12) = 7.509 for APP; F (2, 12) = 47.83 for MBP; ^∗^*p* < 0.05 and ^∗∗∗^*p* < 0.001 vs. Sham group; ^#^*p* < 0.05 vs. ICH group; one-way ANOVA, followed by Turkey's post hoc test. (h) Representative TEM images showing the condition of white matter track in each group. Red arrows indicating demyelination. Scale bars: 2 *μ*m. (i) Quantification of G-ratio from (h). *n* = 3 in each group; F (2, 207) = 48.85; ^∗∗∗^*p* < 0.001 vs. Sham group; ^###^*p* < 0.001 vs. ICH group; one-way ANOVA, followed by Turkey's post hoc test.

Given that the white matter fiber bundles are predominantly responsible for locomotion and motor coordination recovery [29, 30], Western blot assays were performed to assess the white matter damage around hematoma after ICH. The immunoblot bands depicted that the expression of MBP and NF200 was prominently decreased, while that was partially increased with the administration of 5 mg/kg DPX in mice after ICH (Figures 4(a)–4(c)), indicating that hematoma caused WMI and the application of DPX reduced WMI. Meanwhile, the expression of degraded myelin basic protein (dMBP), a symbol of myelin degradation [15], was profoundly elevated in mice after ICH, whereas its expression was, in a degree, downregulated with the application of 5 mg/kg DPX (Figures 4(a) and 4(d)), further confirming that the treatment of DPX protected white matter from hematoma-induced damage. Moreover, the immunostaining images depicted that the optic density of MBP was remarkably decreased, while APP, a maker of damaged axons [15], was obviously increased around hematoma in mice post-ICH (Figures 4(e)–4(g)), whereas this effect was abolished with the addition of 5 mg/kg DPX, to some extent (Figures 4(e)–4(g)). In addition, the TEM was implemented to determine the myelin sheath of white matter fiber bundles, the results depicted that the G-ratio of myelin sheath was clearly increased in group ICH, while it was profoundly decreased with the treatment of 5 mg/kg DPX (Figures 4(h) and 4(i)). Collectively, these results demonstrated that mice suffered from ICH induced WMI, whereas DPX partially reduced WMI around hematoma in mice after ICH.

### 3.3. The Administration of DPX Reduced WMI by Decreasing Iron-Induced ROS Accumulation around Hematoma in Mice after ICH

**Figure 5:**
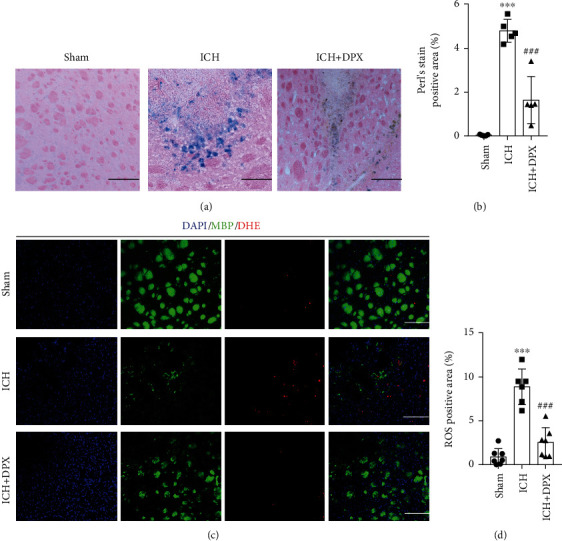
The administration of DPX reduced iron and ROS accumulation after ICH in mice. (a) Representative Perl's blue staining indicating iron accumulation in each group. Scale bars: 50 *μ*m. (b) Semiquantification of iron accumulation from (a). *n* = 5 in each group; F(2, 12) = 61.52; ^∗∗∗^*p* < 0.001 vs. Sham group; ^###^*p* <0.001 vs. ICH group; one-way ANOVA, followed by Turkey's post hoc test. (c) Double staining of MBP (green) and DHE (red) demonstrating the ROS level in each group. Scale bars: 50 *μ*m. (d) The percentage of ROS positive area from (e). *n* = 7 in Sham group, *n* = 6 in ICH group, *n* = 7 in ICH + DPX group; F (2, 17) = 44.08; ^∗∗∗^*p* < 0.001 vs. Sham group; ^###^*p* < 0.001 vs. ICH group; one-way ANOVA, followed by Turkey's post hoc test.

In the light of that oligodendrocytes are susceptible to ROS deposition [14, 15, 31], the iron accumulation was firstly detected using Perls' Blue staining. The representative images delineated that the iron deposition was obviously observed around hematoma in mice after ICH, while it was nearly wiped out with the administration of 5 mg/kg DPX (Figures 5(a) and 5(b)). Furthermore, the double staining of MBP and DHE images showed that the ROS level around hematoma was evidently increased in ICH group, while it was profoundly decreased with the application of 5 mg/kg DPX (Figures 5(c) and 5(d)). These results showcased that surplus iron caused ROS accumulation to minimize WMI around hematoma in mice after ICH.

### 3.4. The Application of DPX Reduced Ferroptosis Resulting from Iron-Induced ROS Accumulation through Upregulating GPX4 and FSP1 around Hematoma in Mice after ICH

**Figure 6:**
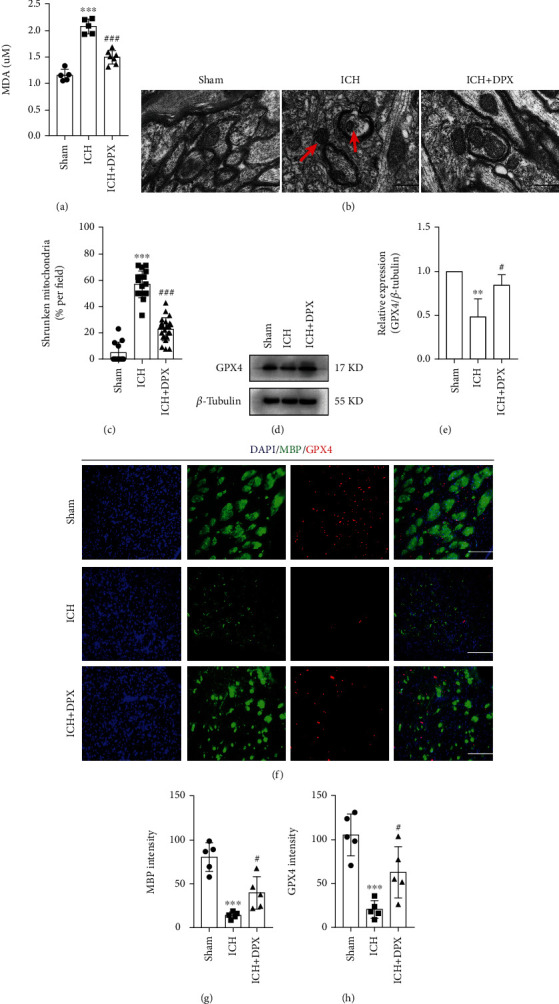
The administration of DPX inhibited glutathione-dependent ferroptosis in oligodendrocytes after ICH in mice. (a) MDA tests indicating the level of lipid peroxidation. n = 5 in Sham group, *n* = 5 in ICH group, *n* = 7 in ICH + DPX group; F (2, 14) = 67.79; ^∗∗∗^*p* < 0.001 vs. Sham group; ^###^*p* < 0.001 vs. ICH group; one-way ANOVA, followed by Turkey's post hoc test. (b) The typical TEM imaging showing the morphology of mitochondria in each group. Red arrow indicating shrunken mitochondria. Scale bars: 2 *μ*m. (c) The percentage of shrunken mitochondria in each group from (b). *n* = 12 in Sham group, *n* = 18 in ICH group, *n* = 23 in ICH + DPX group; Kruskal − Wallis statistic = 42.13; ^∗∗∗^*p* < 0.001 vs. Sham group; ^###^*p* < 0.001 vs. ICH group; one-way ANOVA, followed by Turkey's post hoc test. (d) Immunoblot bands representing the expression of GPX4 in each group. *β*-Tubulin was served as an internal control. (e) Semiquantification of GPX4 from (d). *n* = 3 in each group; F (2, 6) = 11.08; ^∗∗^*p* < 0.01 vs. Sham group; ^#^*p* < 0.05 vs. ICH group; one-way ANOVA, followed by Turkey's post hoc test. (f) Double immunostaining of MBP (green) and GPX4 (red). Scale bars: 50 *μ*m. (g), (h) Semiquantification of the optic intensity of MBP (g) and GPX4 (h). *n* = 5 in each group; F (2, 12) = 26.75 for MBP; F (2, 12) = 17.71 for GPX4; ^∗∗∗^*p* < 0.001 vs. Sham group; ^#^*p* < 0.05 vs. ICH group; one-way ANOVA, followed by Turkey's post hoc test.

The above findings certified that superabundant iron brought about ROS deposition around hematoma after ICH. Considering that ferroptosis is iron-dependent and usually caused by lipid peroxidation [32], the level of lipid peroxidation was then estimated using MDA tests. The results demonstrated that the value of optic density was dramatically elevated, whereas it was obviously lowered with the treatment of 5 mg/kg DPX in perihematoma (Figure 6(a)). At the same time, the TEM was performed to observe the morphology of mitochondria, and the results indicated that the percentage of shrunken mitochondria was profoundly increased in group ICH (Figures 6(b) and 6(c)), while that was clearly downregulated with the addition of 5 mg/kg DPX around hematoma in ICH + DPX group (Figures 6(b) and 6(c)). Furthermore, the expression of glutathione peroxidase 4 (GPX4), a central upstream negative regulator of ferroptosis [33], was significantly downregulated in group ICH, while this effect was partially abrogated with the administration of 5 mg/kg DPX (Figures 6(d) and 6(e)). In addition, the immunostaining of MBP and GPX4 was conducted to assess the expression of GPX4 in oligodendrocytes. The results indicated that the myelination of white matter fiber bundles was greatly reserved in group ICH + DPX, compared with that in group ICH (Figures 6(f) and 6(g)). Meanwhile, the optic density of GPX4 was clearly upregulated with the administration of 5 mg/kg DPX that was remarkably downregulated in mice after ICH (Figures 6(f) and 6(h)). Collectively, these results indicated that DPX inhibited ferroptosis in a glutathione-dependent manner around hematoma in mice after ICH.

**Figure 7:**
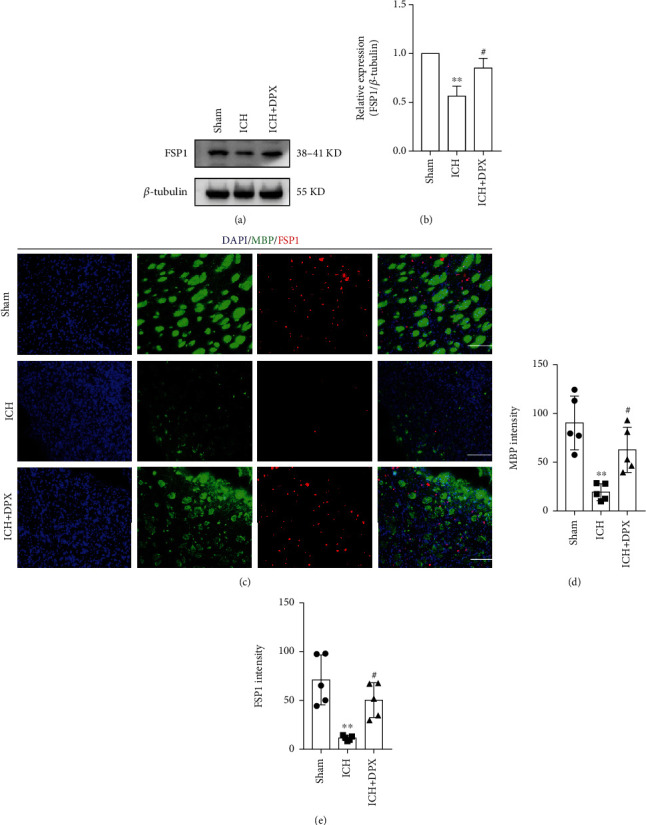
The application of DPX reduced WMI through suppressing glutathione-independent ferroptosis in oligodendrocytes after ICH in mice. (a) Immunoblot bands demonstrating the expression of FSP1 in each group. *β*-Tubulin was served as an internal control. (b) Semi-quantification of FSP1 from (a). *n* = 3 in each group; F (2, 6) = 21.80; ^∗∗^*p* < 0.01 vs. Sham group; ^#^*p* < 0.05 vs. ICH group; one-way ANOVA, followed by Turkey's post hoc test. (c) Double immunostaining of MBP (green) and FSP1 (red). Scale bars: 50 *μ*m. (d), (e) Semiquantification of the optic intensity of MBP (d) and FSP1 (e). *n* = 5 in each group; F (2, 12) = 14.03 for MBP; F (2, 12) = 14.10 for FSP1; ^∗∗^*p* < 0.01 vs. Sham group; ^#^*p* < 0.05 vs. ICH group; one-way ANOVA, followed by Turkey's post hoc test.

Subsequently, the expression of ferroptosis suppressor protein 1 (FSP1), a glutathione-independent ferroptosis-resistance factor [34], was determined using Western blot assays. The immunoblot bands presented that the expression of FSP1 was obviously decreased in group ICH, while that was significantly elevated with the treatment of 5 mg/kg DPX (Figures 7(a) and 7(b)). Moreover, more white matter fiber bundles were reserved in group ICH + DPX than that in group ICH (Figures 7(c) and 7(d)). Meanwhile, the immunostaining of MBP and FSP1 showed that the optic density of FSP1 was higher in group ICH + DPX than that in ICH group (Figures 7(c) and 7(e)). Taken together, these results illustrated that DPX possessed the capacity of inhibiting glutathione-dependent and glutathione-independent ferroptosis around hematoma in mice after ICH.

## 4. Discussion

**Figure 8:**
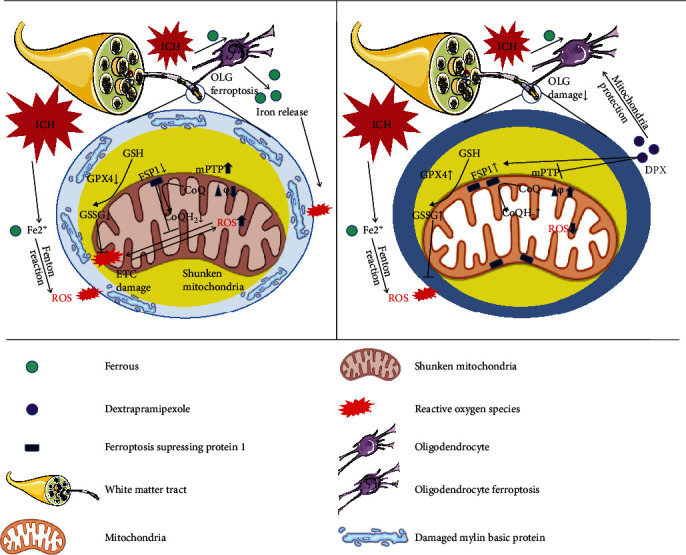
Schematic illustration for the potential therapeutic effects of DPX on ICH and the underlying mechanism.

The basal ganglia, where enriches white matter fiber bundles, is a common site of ICH [35]. ICH usually causes life-long disability that ascribes to severe WMI. The cellular and molecular mechanisms causing WMI is not fully understood. Here, the results demonstrated that iron-induced ROS accumulation initiated ferroptosis resulting in WMI around hematoma, while the administration of DPX reduced iron and ROS deposition to inhibit ferroptosis. With the inhibition of ferroptosis caused by DPX, the WMI was obviously reduced to facilitate locomotion and motor coordination recovery in mice after ICH. Further evidence showed that the effect of DPX on reducing WMI was depending on upregulating the expression of GPX4 and FSP1 (Figure 8). The present study provides a feasible candidate of DPX suppressing ferroptosis to reduce WMI through increasing the expression of GPX4 and FSP1 around hematoma in mice after ICH.

ICH is a devastating disorder that usually brings about life-long neurological impairments in adult [36]. Once ICH occurs, ferrous/ferric iron accumulation around the hematoma contributes to the formation of lethal ROS and lipid peroxidation [9, 37, 38]. The deposition of iron and ROS initiates ferroptosis in both somas and axons at perihematomal site, which is consistent with previous study [39]. And the mitochondria of ferroptotic cells appeared to be shrunken and smaller mitochondrial area when compared with those in sham tissue that is in agreement with previous report [39]. Previous studies have indicated that ferroptosis evokes neuronal damage in aged ICH mice [9, 40]. Here, our results provide evidence that ferroptosis plays an evident role in triggering oligodendrocyte damage after ICH and the administration of DPX mitigates WMI to promote locomotion and motor coordination recovery after ICH. And the underlying mechanism is to upregulating GPX4 and FSP1.

GPX4, the key upstream regulator of ferroptosis [33], maintains the cellular redox homeostasis [41]. GPX4 protects cells against lipid peroxidation damage by transforming highly reactive lipid hydroperoxides (LOOH) to nonreactive lipid alcohols [42]. Thereafter, a negative loop between GPX4 and lipid peroxidation determines the fate of neural cells after injury. Here, GPX4 is downregulated with the presence of elevated lipid peroxidation at perihematomal site after ICH, which is consistent with previous studies [43–46]. Meanwhile, the results also report that GPX4 upregulation induced by the administration of DPX alleviates lipid peroxidation injury to neural cells after ICH, which is in line with previous studies that elevated GPX4 expression mitigates ICH-induced acute brain injury by activating ferroptosis [44, 46, 47]. To our limited knowledge, it is the first work to provide a rationale that DPX inhibits ferroptosis through increasing the expression of GPX4, which provides a rationale administrating DPX for the treatment of ICH targeting ferroptosis.

Furthermore, FSP1, a glutathione-independent ferroptosis suppressor, regulates the cellular redox balance via FSP1/NAD (P) H/coenzyme Q10 (CoQ10) axis that is in parallel with the representative GPX4 pathway to repress ferroptosis [34]. Here, the results illustrate that the expression of FSP1 is slightly upregulated around hematoma after ICH in comparison with Sham. The reason for this phenomenon might ascribe to a self-negative feedback regulation that is activated to prevent lipid peroxidation and ferroptosis, which is dramatically increased after ICH. Further evidence reveals that the expression of FSP1 is remarkably increased with the treatment of DPX, supporting the notion that DPX inhibits ICH-induced ferroptosis through increasing the expression of FSP1.

DPX, also known as KNS-760704 or (6R)-4,5,6,7-tetrahydro-N6-propyl-2,6-benzothiazole-diamine, is the R-isomer of the antiparkinsonian drug pramipexole, with low affinity to bind dopamine receptors but holding the ability of improving mitochondrial efficiency through F1Fo-ATP synthase [48, 49]. Meanwhile, DPX has been administrated to treat amyotrophic lateral sclerosis (ALS) and hypereosinophilic syndromes in clinic [16, 50]. Currently, DPX has been applied to treat ischemic brain injury, and the results indicate that DPX increases mitochondrial ATP production to reduce energy failure and prevents intracellular Ca^2+^ overload in neurons and astrocytes [19]. Most recent study shows that DPX upregulates mitophagy via mediating both PTEN-induced putative kinase1 (PINK1) and Parkin-dependent mechanisms to protect myocardial cells from ischemic reperfusion injury [51]. Here, the results showcase that the administration of DPX reduces ROS accumulation around hematoma, which is agreement with previous study [22, 52, 53]. Meantime, the application of DPX reduces iron deposition; the reason might contribute to regulating the expression of iron-transport proteins. Our previous study demonstrates that the expression of divalent metal transporter 1 (DMT1) is increased with excessive iron deposition in injured spinal cord, and reduced DMT1 expression decreases iron accumulation with the treatment of deferoxamine [54]. Further evidence indicates that the upregulation of transferrin receptor 1 (TfR1) reduces iron accumulation with the application of apoferritin [55]. Hence, it is reasonable to believe that DPX reduces iron accumulation at perihematomal site that might ascribe to mediating the expression of DMT1 and TfR1 that is going to be investigated in our future research.

## 5. Conclusions

In conclusion, the present study provides a rationale that ferroptosis plays a significant role in WMI resulting from iron and ROS accumulation around hematoma, and DPX decreases iron and ROS accumulation to suppress ferroptosis through upregulating of GPX4 and FSP1. With the inhibition of ferroptosis, the WMI is alleviated to maintain the integrity of white matter fiber bundles in basal ganglia, thereafter facilitating locomotion and motor coordination recovery in mice after ICH. Collectively, the present study uncovers the underlying mechanism and elucidates the therapeutic effect of DPX on ICH, and even in other central nervous system (CNS) diseases with the presence of ferroptosis.

## Data Availability

The data used to support the findings of this study are available from the corresponding author upon reasonable request.
